# Supplementary material for: Scalable multiple whole-genome alignment and locally collinear block construction with SibeliaZ
Source: Nat Commun. 2020 Dec 10;11:6327. doi: 10.1038/s41467-020-19777-8 (PMC7728760; doi:10.1038/s41467-020-19777-8)
Supplement: Supplementary file 1 — Supplementary Information [file 41467_2020_19777_MOESM1_ESM.pdf]

Scalable multiple whole-genome alignment and locally collinear block construction with SibeliaZ
Supplementary information

Minkin et al

Supplementary Note 1: Parameter details and command lines

We tried to find the optimal parameters for all tools. For Sibelia, which could only run on simulated data, we used the parameter set designed to yield the highest sensitivity (called the "far" set in Sibelia). Progressive Cactus requires a phylogenetic tree in addition to the input genome which it uses for adjusting the internal parameters. For the simulated datasets, we used the real tree generated by the simulator; for the mice genomes, we used the guide tree from .[1] Multiz+TBA were run with default parameters since its documentation does not provide a clear guideline on how to adjust the parameters according to evolutionary distances between the input sequences. We could not compile the version of MultiZ+TBA publicly available for download and used a slightly modified version provided by Robert S. Harris. For TwoPaCo and spoa, we set the parameters following the guidelines provided with the respective software. SibeliaZ was run with $k = 25$, $b = 200$, $m = 50$, and $a = 150$.

We performed all experiments on a machine running Ubuntu 16.04.3 LTS with 512 GB of RAM and a 64 core CPU Intel Xeon CPU E5-2683 v4. We were limited to using at most 32 threads at any given time. Progressive Cactus was run with 32 threads, since the authors recommended to use as many threads as possible for the best performance. MultiZ+TBA and Sibelia are both single-threaded. (There were several submissions to Alignathon which used an extensively parallelized MultiZ or TBA; unfortunately, the software packages used for those submissions are not available publicly for download.) TwoPaCo and SibeliaZ-LCB were run 16 and 32 threads respectively. We note that spoa is run on each block, and our software includes a wrapper to automate this.

Here are the exact command lines for the tools we ran.

TwoPaCo:

```
twopaco -k <k_value> -f <bloom_filter_size> -t 16 -o <dbg_graph> <genomes_file>
```

SibeliaZ-LCB:

```
SibeliaZ-LCB --fasta <genomes_file> --graph <dbg_graph> -o <output_directory>
-k 25 -b 200 -m 50 -a 150 -t 4
```

spoa:

```
spoa <input_fasta_file> -l 1 -r 1 -e 8
```

Sibelia:

```
Sibelia <genomes_file> -o <output_directory> -s far --lastk 50 -m 50 --nopostprocess
```

MultiZ:

```
all_bz <guide_tree>
tba <guide_tree> *.*.maf <outputMafFile>
```

Progressive Cactus:

```
runProgressiveCactus.sh --maxThreads 32 <seqFile> <workDir> <outputHalFile>
source ./environment && hal2mafMP.py <outputHalFile> <outputMafFile>
```

All running times and memory usage numbers were obtained using the GNU time utility. The exact versions of the software are in Table 4.

Supplementary Note 2: Simulation details

We used small simulated data in order to understand the role of a dataset's genomic distance and of our parameter settings. We used ALF[2] for simulation because it simulates point mutations as well as genome-wide events such as inversions, translocations, fusions/fissions, gene gain/loss, and lateral gene transfer. Furthermore, ALF is useful for benchmarking as it also produces an alignment which represents the true homology between the genomes, making it possible to directly assess the precision and recall. We simulated 6 datasets, each one consisting of 10 bacterial genomes. Each genome is composed of 1500 genes and of size approximately 1.5 Mbp. We used such relatively small datasets to allow us to efficiently explore the parameter space. Each of the 6 datasets corresponded to a different parameter for distance from the root to leaf species, which we varied from 0.03 to 0.18 substitutions per site with the step of 0.03. In ALF, different proteins evolve with different rates, which are derived from the base value using a probabilistic distribution. See[2] for more details and the simulation recipes for the exact values of the parameters. For genome-wide events, we used ALF's default rates. Links to download the the simulation parameter files, the simulated genomes, and their alignments are available at the GitHub repository (see "Data availability" section in the main paper).

Supplementary Note 3: Other related work

A closely related problem to multiple whole-genome alignment is synteny reconstruction. In this setting, genomes are decomposed into large blocks such that the gene order within each block is preserved. This is similar to locally collinear blocks, but collinear blocks are usually smaller blocks representing single genes or exons (or non-coding DNA). Collinear blocks can be viewed as high resolution synteny blocks and, in general, the distinction between the two concepts can be blurry. For a discussion on representation of synteny blocks at multiple scales, see.[3] Synteny blocks are often reconstructed from anchors such as genes,[4–7] locally-collinear blocks[8] and, less commonly, from the nucleotide sequences directly.[3,9]

A related active research area is data structures for representing pan-genomes.[10] A pan-genome as a collection of related genomes that are to be analyzed jointly. For a review on computational pan-genomics, see.[11–13] Constructing a data structure for the efficient storage and querying of a pan-genome is related but tangential to the problem of identifying collinear blocks, which we consider in this paper. Pan-genome data structures are concerned with efficiently representing the homology within the pan-genome, while we focus on fast algorithms for obtaining such homologies. There is naturally some overlap between the two areas, e.g. some pan-genome tools include a multiple whole-genome alignment component.[14,15] Others use the de Bruijn graph for representing the pan-genome.[16–19] Our approach also relies on a de Bruijn graph, though we use it as a technique to find collinear blocks rather than to represent them.

Supplementary Tables and Figures

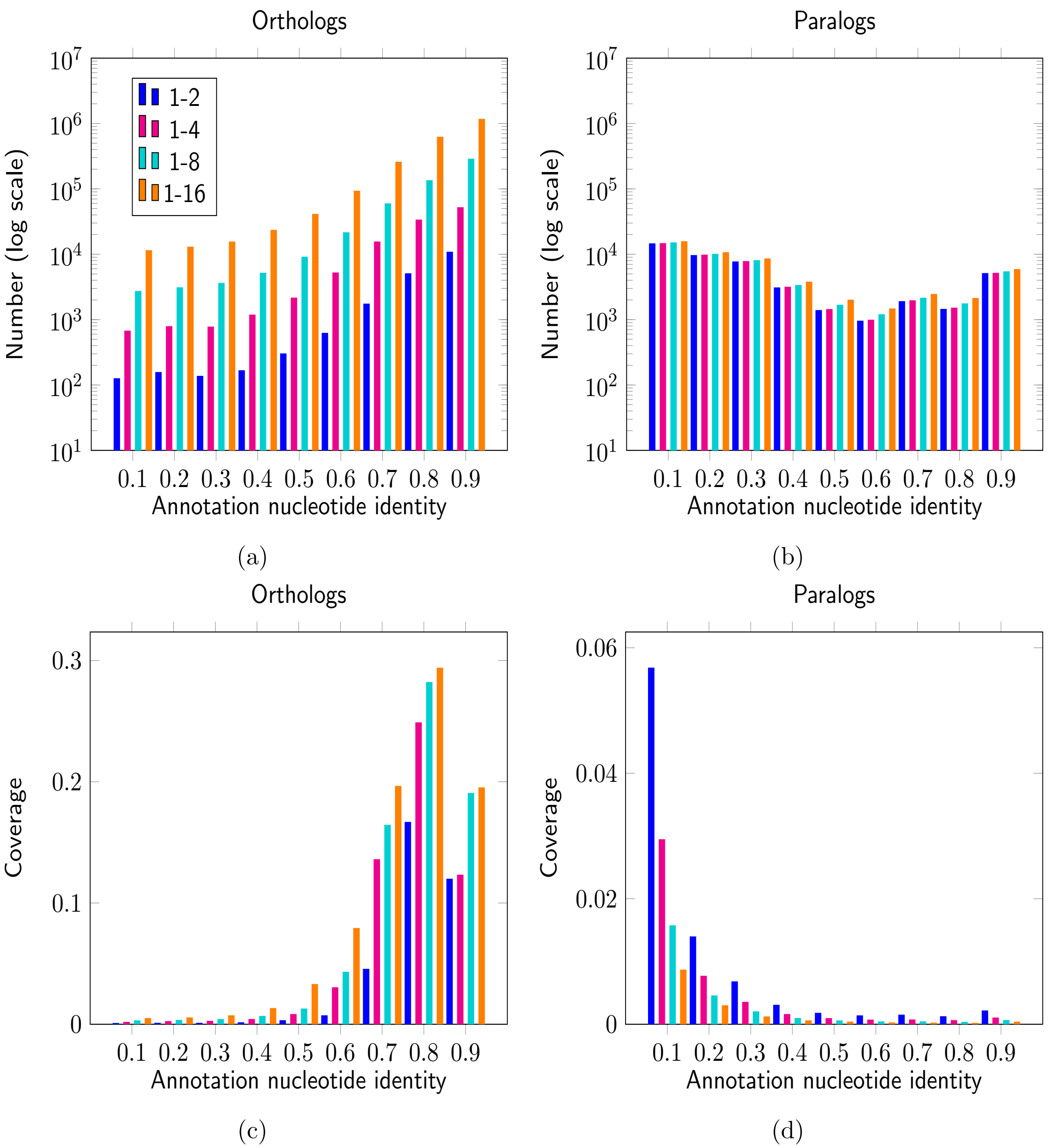

Supplementary Figure 1: Properties of the pairwise alignments constructed from pairs of homologous protein-coding genes in the various mice datasets. Panels (a) and (b) show the total number of genes for each nucleotide identity value for orthologous and paralogous pairs respectively; (c) and (d) demonstrate coverage of the genomes by these two categories of the genes.

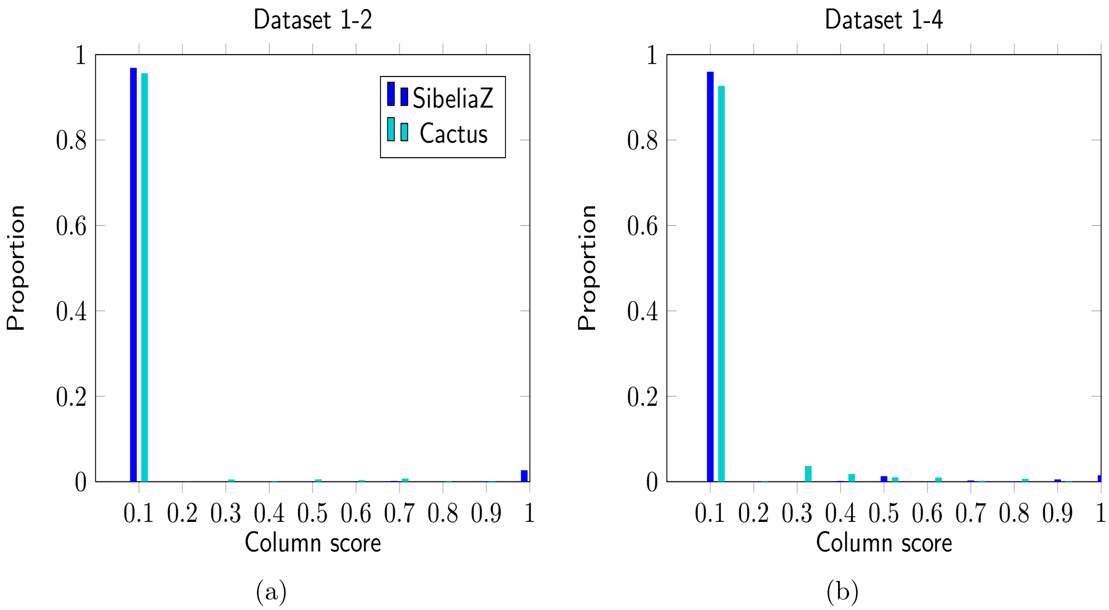

Supplementary Figure 2: Histogram of the average number of nucleotide differences $\pi(c)$ calculated for each column of SibeliaZ's and Cactus' alignment, for datasets 1-2 (a) and 1-4 (b) consisting of 2 and 4 mice geneomes respectively.

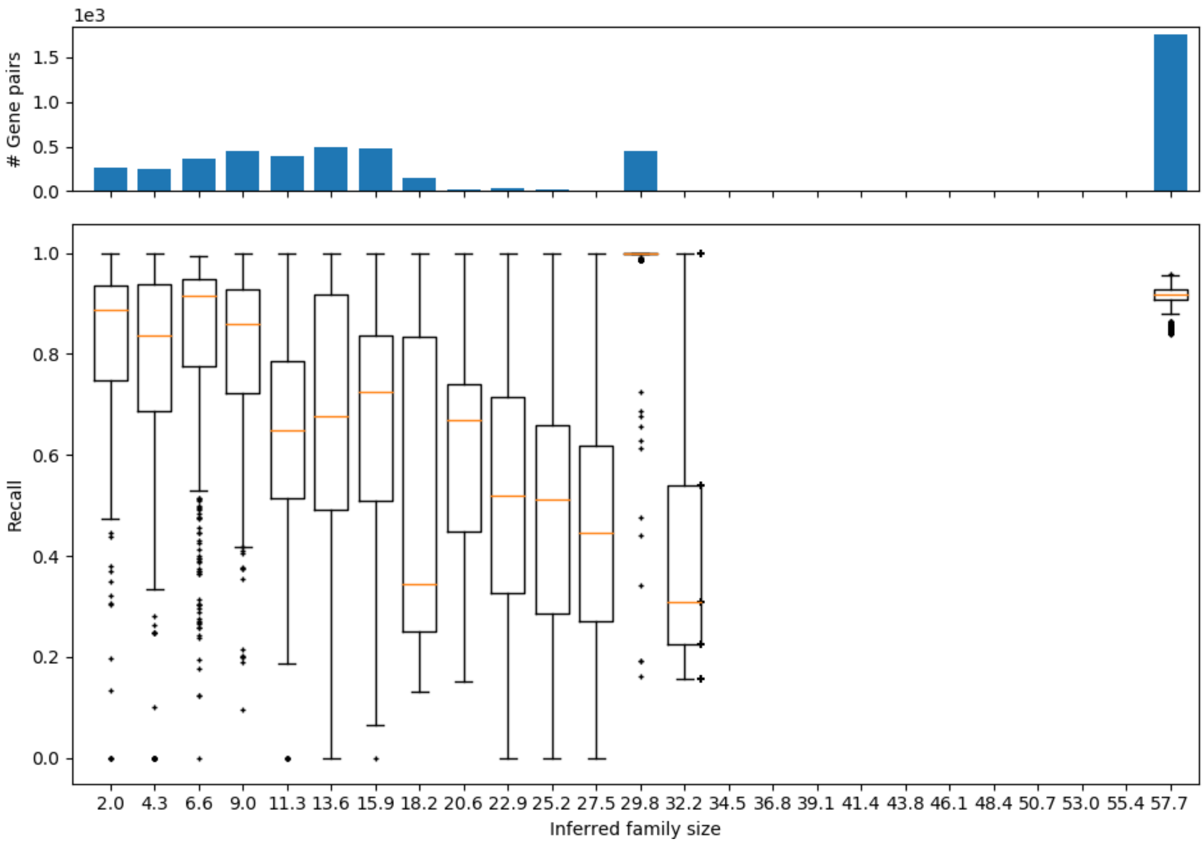

Supplementary Figure 3: The recall of paralogous genes by SibeliaZ as a function of inferred family size, using the two-mice dataset. The $n = 5152$ gene pairs were split into 25 equally-sized disjoint bins based on the inferred family size. The top histogram shows the number of gene pairs in each bin. Exact sizes for each bin are (267, 250, 360, 452, 396, 489, 481, 155, 24, 35, 21, 12, 450, 5, 0, 0, 0, 0, 0, 0, 0, 0, 0, 0, 1755). Points belonging to non-empty bins of size less than 10 are shown individually. Each box plot shows the median (middle line), the interquartile range (outer borders of the box), minimum and maximum values within ± 1.5 of the interquartile range (whiskers). Data points outside of the ± 1.5 interquartile range are represented by individual data points.

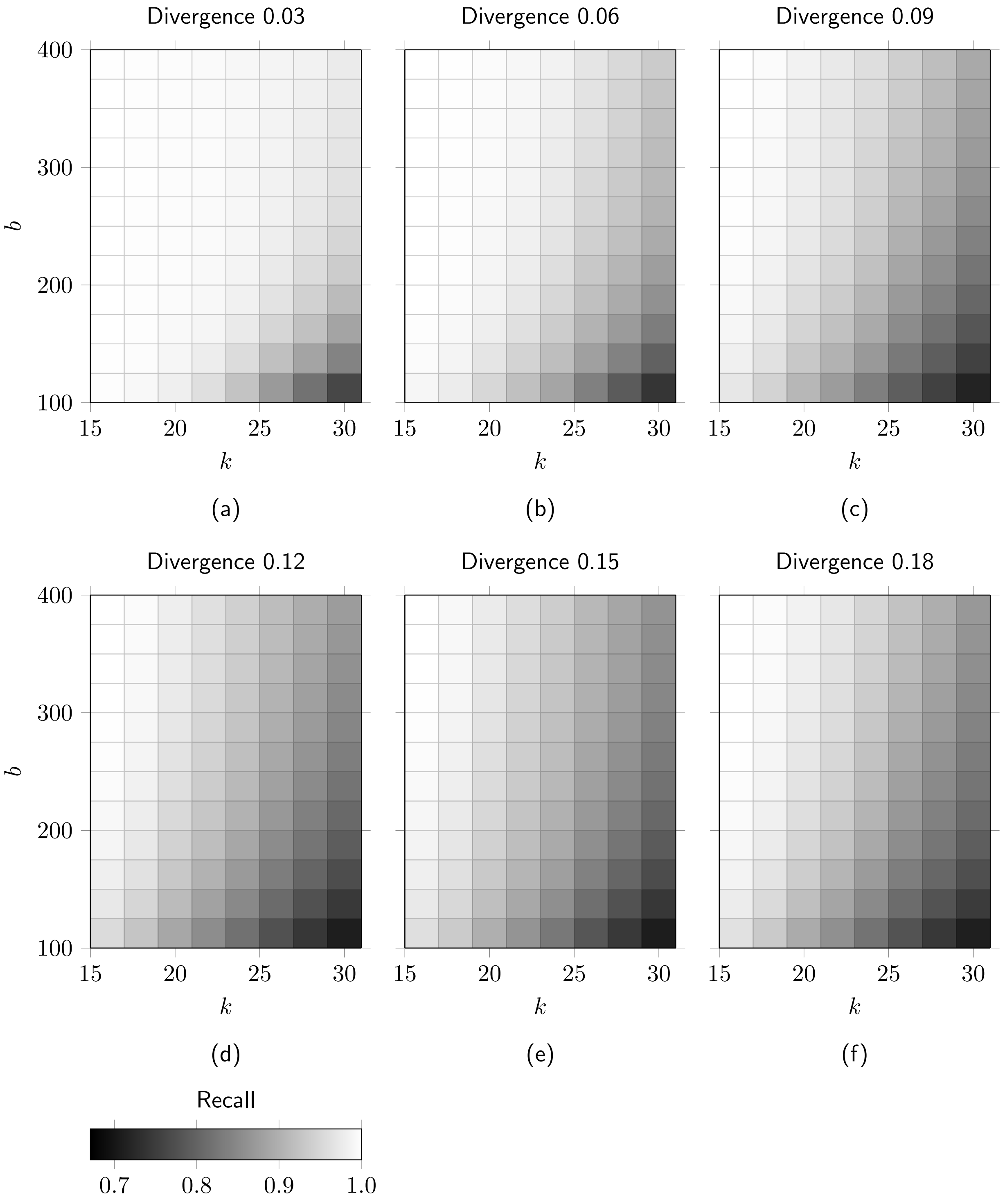

Supplementary Figure 4: Effects of the parameters k and b on recall. Each panel (a-f) contains a heatmap corresponding to a simulated dataset with the specified root-to-leaf divergence in substitutions per site and a cell corresponds to a combination of parameters.

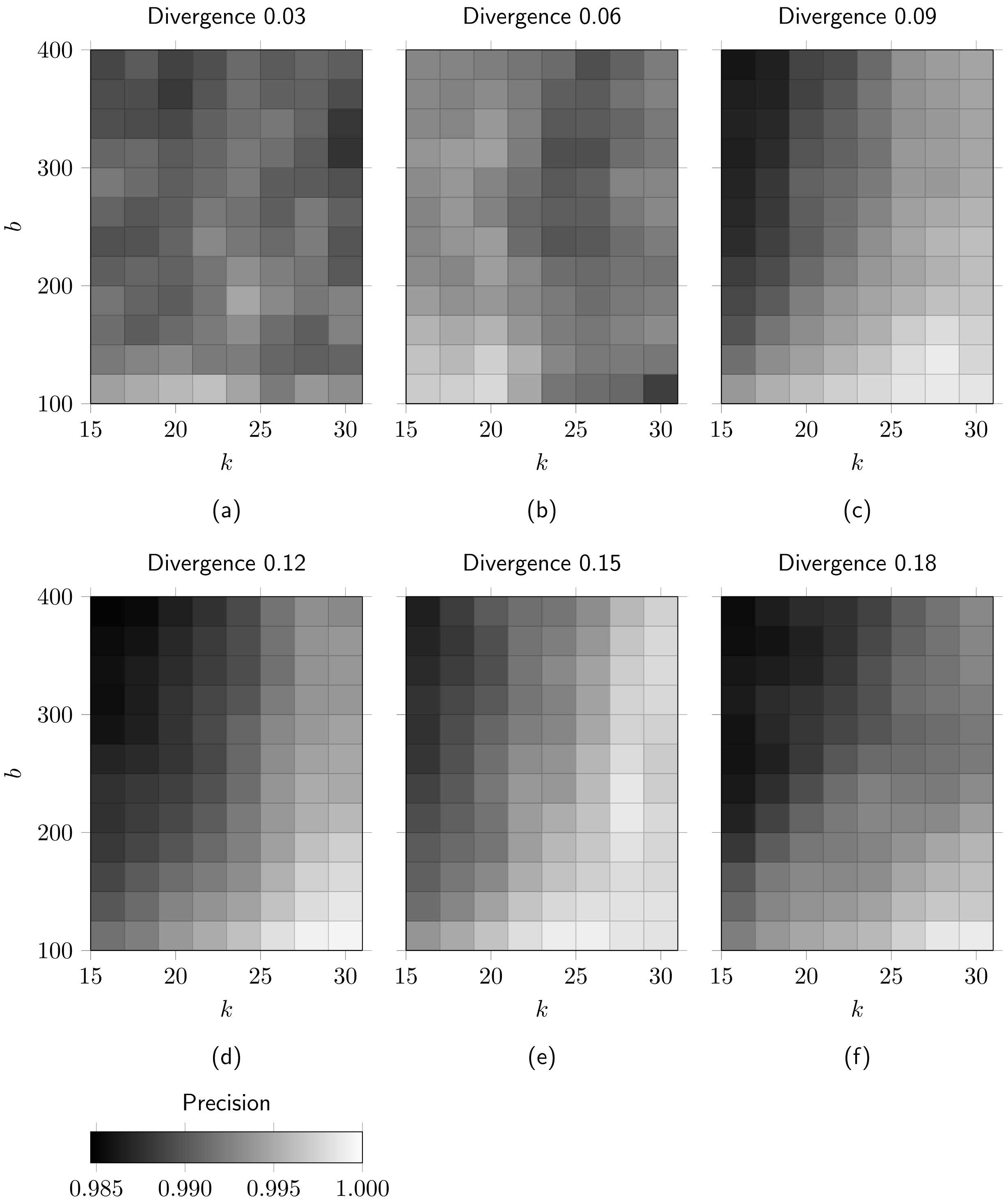

Supplementary Figure 5: Effects of the parameters k and b on precision. Each panel (a-f) contains a heatmap corresponding to a simulated dataset with the specified root-to-leaf divergence in substitutions per site and a cell corresponds to a combination of parameters.

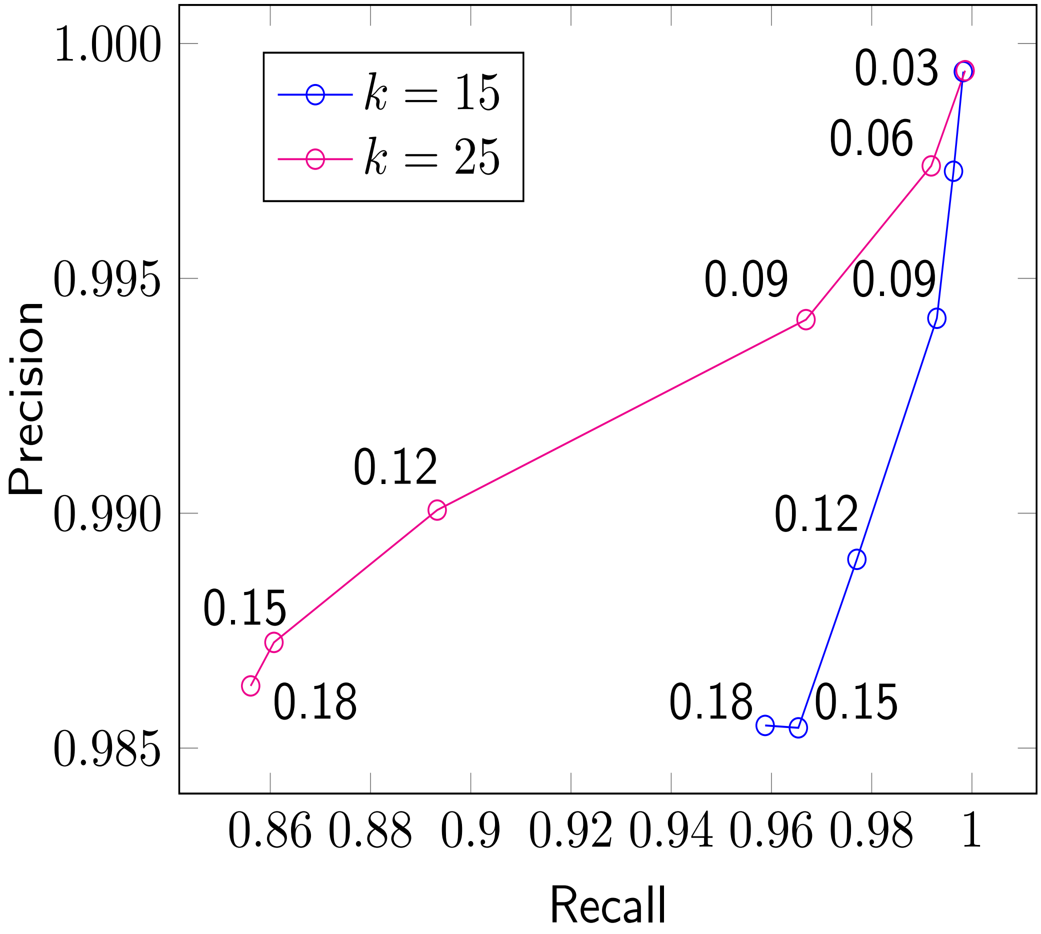

Supplementary Figure 6: The accuracy of SibeliaZ as a function of genomic divergence. Each point is labeled with the height of the phylogenetic tree (in terms of substitutions per site) of its respective simulated dataset.

ID	Strain	Size (Mb)	N. Scaffolds
1	C57BL/6J	2,819	336
2	129S1/SvImJ	2,733	7,154
3	A/J	2,630	4,688
4	AKR/J	2,713	5,953
5	CAST/EiJ	2,654	2,977
6	CBA/J	2,922	5,466
7	DBA/2J	2,606	4,105
8	FVB/NJ	2,589	5,013
9	NOD/ShiLtJ	2,982	5,544
10	NZO/HiLtJ	2,699	7,022
11	PWK/PhJ	2,560	3,140
12	WSB/EiJ	2,690	2,239
13	BALB/cJ	2,627	3,825
14	C57BL/6NJ	2,807	3,894
15	C3H/HeJ	2,701	4,069
16	LP/J	2,731	3,499

Supplementary Table 1: Properties of the assembled mice genomes available at GenBank.

Dataset	SibeliaZ/TwoPaCo	SibeliaZ/SibeliaZ-LCB	SibeliaZ/spoa	SibeliaZ/Total	Cactus
1-2	12 (9.30)	74 (36.00)	68 (121.50)	154 (121.50)	2,279 (37.50)
1-4	25 (17.70)	96 (72.70)	115 (133.60)	236 (133.60)	6,105 (89.80)
1-8	49 (34.50)	104 (106.30)	240 (132.50)	393 (132.50)	-
1-16	101 (68.10)	153 (183.60)	736 (133.50)	990 (183.60)	-

Supplementary Table 2: Running time (minutes) and memory usage (gigabytes, in parenthesis) of SibeliaZ and Cactus on the mice datasets. A dash in a column indicates that the program did not complete within in a week.

Dataset	SibeliaZ Recall	Cactus Recall	SibeliaZ Precision	Cactus Precision
1-2	0.95	0.97	0.93	0.89
1-4	0.95	0.92	0.96	0.90

Supplementary Table 3: Recall and precision of SibeliaZ and Cactus. We used pairwise local alignments of chromosomes 1 from the different datasets produced by LASTZ as the ground truth.

Software	Version	Repostiory	Commit
Sibelia	3.0.7	bioinf/Sibelia	397e6877116006c8591cbe14a7c6d366d1e0751a
SibeliaZ	1.2.0	medvedevgroup/SibeliaZ	e90f5b25c931b5b011b98c558670f1697334ef69
TwoPaCo	0.9.4	medvedevgroup/TwoPaCo	9b9fee321dd561b7bd2b18892b0b2653c58eb6dd
spoa	3.0.1	rvaser/spoa	4c87d6831e9898dcaf2830182afece85e77b09ce
Progressive Cactus	0.0	glennhickey/progressiveCactus	c4bed56c0cd48d23411038acb9c19bcae054837e
ALF	0.97	DessimozLab/ALF	7dfed367bd1f5c002dbbd2a23d597638b36b379c
LASTZ	1.04.00	lastz/lastz	ce6e5f598e3e2190b23c512e571b9f9c244adb6e
LAGAN	2.0	-	-
MULTIZ	11.2	-	-

Supplementary Table 4: GitHub revisions of the software we used. We used the most up-to-date versions available at the time of development of our project. A dash indicates that we downloaded the software from the author's website.

Supplementary References

[1] Lilue, J. *et al.* Sixteen diverse laboratory mouse reference genomes define strain-specific haplotypes and novel functional loci. *Nature Genetics* **50**, 1574 (2018).

[2] Dalquen, D. A., Anisimova, M., Gonnet, G. H. & Dessimoz, C. ALF – A Simulation Framework for Genome Evolution. *Molecular Biology and Evolution* **29**, 1115–1123 (2011).

[3] Minkin, I., Patel, A., Kolmogorov, M., Vyahhi, N. & Pham, S. Sibelia: A scalable and comprehensive synteny block generation tool for closely related microbial genomes. In Darling, A. & Stoye, J. (eds.) *Algorithms in Bioinformatics*, 215–229 (Springer Berlin Heidelberg, Berlin, Heidelberg, 2013).

[4] Pevzner, P. & Tesler, G. Genome rearrangements in mammalian evolution: lessons from human and mouse genomes. *Genome Research* **13**, 37–45 (2003).

[5] Ng, M.-P. *et al.* Orthoclusterdb: an online platform for synteny blocks. *BMC Bioinformatics* **10**, 192 (2009).

[6] Pham, S. & Pevzner, P. Drimm-synteny: decomposing genomes into evolutionary conserved segments. *Bioinformatics* **26**, 2509–2516 (2010).

[7] Proost, S. *et al.* i-adhore 3.0 – fast and sensitive detection of genomic homology in extremely large data sets. *Nucleic Acids Research* **40**, e11–e11 (2011).

[8] Kolmogorov, M. *et al.* Chromosome assembly of large and complex genomes using multiple references. *Genome Research* **28**, 1720–1732 (2018).

[9] Doerr, D. & Moret, B. M. Sequence-based synteny analysis of multiple large genomes. In *Comparative Genomics*, 317–329 (Springer, 2018).

[10] Tettelin, H. *et al.* Genome analysis of multiple pathogenic isolates of streptococcus agalactiae: implications for the microbial pan-genome. *Proceedings of the National Academy of Sciences of the United States of America* **102**, 13950–13955 (2005).

[11] Vernikos, G., Medini, D., Riley, D. R. & Tettelin, H. Ten years of pan-genome analyses. *Current Opinion in Microbiology* **23**, 148–154 (2015).

[12] Marschall, T. *et al.* Computational pan-genomics: status, promises and challenges. *Briefings in Bioinformatics* **19**, 118–135 (2018).

[13] Zekic, T., Holley, G. & Stoye, J. Pan-genome storage and analysis techniques. In *Comparative Genomics*, 29–53 (Springer, 2018).

[14] Ernst, C. & Rahmann, S. Pancake: a data structure for pangenomes. In *OASIcs-OpenAccess Series in Informatics*, vol. 34 (Schloss Dagstuhl-Leibniz-Zentrum fuer Informatik, 2013).

[15] Laing, C. *et al.* Pan-genome sequence analysis using panseq: an online tool for the rapid analysis of core and accessory genomic regions. *BMC Bioinformatics* **11**, 461 (2010).

[16] Marcus, S., Lee, H. & Schatz, M. C. Splitmem: a graphical algorithm for pan-genome analysis with suffix skips. *Bioinformatics* **30**, 3476–3483 (2014).

[17] Holley, G., Wittler, R. & Stoye, J. Bloom filter trie: an alignment-free and reference-free data structure for pan-genome storage. *Algorithms for Molecular Biology* **11**, 3 (2016).

[18] Beller, T. & Ohlebusch, E. A representation of a compressed de bruijn graph for pan-genome analysis that enables search. *Algorithms for Molecular Biology* **11**, 20 (2016).

[19] Sheikhizadeh, S., Schranz, M. E., Akdel, M., de Ridder, D. & Smit, S. Pantools: representation, storage and exploration of pan-genomic data. *Bioinformatics* **32**, i487–i493 (2016).
