# Supplementary material for: Scalable multiple whole-genome alignment and locally collinear block construction with SibeliaZ
Source: Nat Commun. 2020 Dec 10;11:6327. doi: 10.1038/s41467-020-19777-8 (PMC7728760; doi:10.1038/s41467-020-19777-8)
Supplement: Supplementary file 2 — Reporting Summary [file 41467_2020_19777_MOESM2_ESM.pdf]

nature research

Corresponding author(s): Ilia Minkin

Last updated by author(s): 9/20/2020

Reporting Summary

Nature Research wishes to improve the reproducibility of the work that we publish. This form provides structure for consistency and transparency in reporting. For further information on Nature Research policies, see our Editorial Policies and the Editorial Policy Checklist.

Statistics

For all statistical analyses, confirm that the following items are present in the figure legend, table legend, main text, or Methods section.

n/a	Confirmed	
[ ]	[x]	The exact sample size (*n*) for each experimental group/condition, given as a discrete number and unit of measurement
[ ]	[x]	A statement on whether measurements were taken from distinct samples or whether the same sample was measured repeatedly
[x]	[ ]	The statistical test(s) used AND whether they are one- or two-sided<br>*Only common tests should be described solely by name; describe more complex techniques in the Methods section.*
[x]	[ ]	A description of all covariates tested
[x]	[ ]	A description of any assumptions or corrections, such as tests of normality and adjustment for multiple comparisons
[x]	[ ]	A full description of the statistical parameters including central tendency (e.g. means) or other basic estimates (e.g. regression coefficient) AND variation (e.g. standard deviation) or associated estimates of uncertainty (e.g. confidence intervals)
[x]	[ ]	For null hypothesis testing, the test statistic (e.g. *F*, *t*, *r*) with confidence intervals, effect sizes, degrees of freedom and *P* value noted<br>*Give P values as exact values whenever suitable.*
[x]	[ ]	For Bayesian analysis, information on the choice of priors and Markov chain Monte Carlo settings
[x]	[ ]	For hierarchical and complex designs, identification of the appropriate level for tests and full reporting of outcomes
[x]	[ ]	Estimates of effect sizes (e.g. Cohen's *d*, Pearson's *r*), indicating how they were calculated

Our web collection on statistics for biologists contains articles on many of the points above.

Software and code

Policy information about availability of computer code

Data collection	ALF 0.97 (to generate the simulated dataset)
Data analysis	Sibelia 3.0.7<br>SibeliaZ 1.2.0<br>TwoPaCo 0.9.4<br>spoa 3.0.1<br>Progressive Cactus 0.0<br>LASTZ 1.04.00<br>LAGAN 2.0<br>MULTIZ 11.2

For manuscripts utilizing custom algorithms or software that are central to the research but not yet described in published literature, software must be made available to editors and reviewers. We strongly encourage code deposition in a community repository (e.g. GitHub). See the Nature Research guidelines for submitting code & software for further information.

Data

Policy information about availability of data

All manuscripts must include a data availability statement. This statement should provide the following information, where applicable:

- Accession codes, unique identifiers, or web links for publicly available datasets
- A list of figures that have associated raw data
- A description of any restrictions on data availability

C57BL/6J GCA_000001635.8
129S1/SvImJ GCA_001624185.1
A/J GCA_001624215.1
AKR/J GCA_001624295.1
CAST/EiJ GCA_001624445.1
CBA/J GCA_001624475.1
DBA/2J GCA_001624505.1
FVB/NJ GCA_001624535.1
NOD/ShiLtJ GCA_001624675.1
NZO/HiLtJ GCA_001624745.1
PWK/PhJ GCA_001624775.1
WSB/EiJ GCA_001624835.1
BALB/cJ GCA_001632525.1
C57BL/6NJ GCA_001632555.1
C3H/HeJ GCA_001632575.1
LP/J GCA_001632615.1

Table above contains the list of GenBank accession numbers of the mice genomes. The nine simulated datasetswe generated (Supplementary Figures 4, 5, 6), ground-truth alignments for the mouse data(Figure 4, Supplementary Figures 1, 2, 3), and alignments produced by SibeliaZ and Progressive Cactus (Figure 4, Supplementary Figures 2, 3) are available for download athttps://github.com/medvedevgroup/SibeliaZ/blob/master/DATA.txt.

Field-specific reporting

Please select the one below that is the best fit for your research. If you are not sure, read the appropriate sections before making your selection.

- [x] Life sciences
- [ ] Behavioural & social sciences
- [ ] Ecological, evolutionary & environmental sciences

For a reference copy of the document with all sections, see nature.com/documents/nr-reporting-summary-flat.pdf

Life sciences study design

All studies must disclose on these points even when the disclosure is negative.

Sample size	The main experiment in our paper involved computing alignment of 16 mice genomes and then comparing it against 2263940 pairs of orthologous genes and 53050 pairs of paralogous genes. We did not use statistical methods to calculate the sample size. We chose the sample size based on the fact that a dataset of such size was going to pose a significant challenge for any state-of-the-art whole genome aligner.
Data exclusions	We removed any pairs of paralogous genes with overlapping coordinates, as these were likely misannotations, as confirmed by Ensembl helpdesk.
Replication	We verified the accuracy of our method on several simulated datasets.
Randomization	We did not randomize the data we used since we did not aim to evaluate an intervention and comparing a treatment and a control group.
Blinding	Blinding is not relevant to our study since it does not involve human participants.

Reporting for specific materials, systems and methods

We require information from authors about some types of materials, experimental systems and methods used in many studies. Here, indicate whether each material, system or method listed is relevant to your study. If you are not sure if a list item applies to your research, read the appropriate section before selecting a response.

Materials & experimental systems

n/a	Involved in the study
☒	☐ Antibodies
☒	☐ Eukaryotic cell lines
☒	☐ Palaeontology and archaeology
☒	☐ Animals and other organisms
☒	☐ Human research participants
☒	☐ Clinical data
☒	☐ Dual use research of concern

Methods

n/a	Involved in the study
☒	☐ ChIP-seq
☒	☐ Flow cytometry
☒	☐ MRI-based neuroimaging
